# Comment on *Informing relatives of their genetic risk: an examination of the Belgian context*

**DOI:** 10.1038/s41431-022-01066-1

**Published:** 2022-03-01

**Authors:** Aisling de Paor

**Affiliations:** School of Law and Government, Dublin City University, Glasnevin, Dublin 9, Ireland

**Keywords:** Ethics, Social sciences

## Introduction

In 2022, the European Journal of Human Genetics published an article by Amicia Philips, Thomas Bronselaer, Pascal Borry, Ine Van Hoyweghen, Danya F Vears, Laurent Pasquier and Stefaan Callens: ‘Informing relatives of their genetic risk: an examination of the Belgian legal context’ [1]. With an original and thought provoking insight into one of the most topical and relevant medico-legal challenges arising with the advent of a new genetic era, this article offers a well-informed and accessible analysis of the many ethical and legal issues presented in this area, as well as the practical questions arising for physicians in medical practice. It has relevance and application across disciplines (including law, medicine, ethics and sociology) and across various jurisdictions. This comment will provide some context to this article and highlight the key points of analysis.

Rapid advances in genetic science and new genetic technologies have led to new medical opportunities in detecting and indicating predisposition to disease and disability. These advances are revolutionising the practice of medicine, as well as the diagnosis, treatment, and increasingly the prevention of disease. Technology such as genetic testing provides insights into one’s future health; it can detect what genes an individual may have, thereby acting as a predictive tool in determining whether an individual may develop certain conditions and diseases. Genetic testing similarly reveals such insights about one’s blood relatives. Alongside this new world of diagnostic based genetic medicine, a minefield of ethical dilemmas and (challenging) legal and policy questions are presented, particularly around the use and misuse of genetic information, as well as around the disclosure and non-disclosure of genetic information by medical professionals and other third parties.

In this context, the unique nature of genetic information is noteworthy; it is particularly personal, sensitive and predictive. The information is familial information, which therefore expands the scope of those whose interests and rights are at stake and who are potentially vulnerable to harm as result of non-disclosure of the information. Given these advances and the familial nature of genetic information, medical professionals now have a growing knowledge of valuable and predictive medical data, about a patient and also their family members. The concept of the ‘patient’ is therefore evolving. The disclosure of such genetic information and risk profile can allow for more refined monitoring and screening of one’s health, as well as facilitating psychological, emotional, financial preparedness. The information allows individuals to alter their diet or lifestyle, where necessary. Where preventative measures are available for diseases, there is added incentive to know one’s genetic make up. This provokes questions around protecting privacy rights of patients and safeguarding the long established duty of confidentiality, as well as a corresponding desire to warn family members about their genetic risk and therefore prevent harm to them. It raises the question of whether and how to communicate familial genetic risk beyond the traditional doctor–patient relationship (particularly in circumstances with strained family dynamics or where a patient refuses to consent to such disclosure). The familial and shared nature of genetic information therefore points to a conflict of rights between a patient’s right to privacy and confidentiality and a family member’s potential right to know. It leaves medical professionals in a difficult position in trying to balance or reconcile these competing rights and to ascertain whose rights should take precedence. Determining best practice around the non-consensual disclosure of genetic information has been particularly challenging in medical settings, as well as in a legal context.

## Comparative benchmarks and genetic risk disclosure

Emerging professional guidelines, law and policy and case law around the world have been slowly responding to these challenges and recognising a varying duty on the part of medical professionals and patients to prevent harm to at- risk relatives by disclosing genetic information and breaching the duty of confidentiality, where necessary.

Following a thought-provoking introduction which sets the scene and highlights the key issues arising, this article focuses on Belgium and examines existing Belgian legislation. In Belgium, there is no specific law or professional guidance regulating the permissibility of disclosure of genetic information without a patient’s consent. This article examines whether existing legal frameworks might apply in these circumstances. In this respect, it considers the relevant legal frameworks regarding three key considerations: (1) patients’ duties to family members, (2) respect for patient confidentiality and privacy, and (3) health care professionals’ duties to family members. Against this context and framework, the article also examines emerging international benchmarks in this area and considers how such international precedent might inform the interpretation of the existing Belgian law.

The long recognised and safeguarded duty of patient confidentiality and right to privacy (which is legally recognised in Belgian law and European law) is a cornerstone of medical practice; it provides security and privacy for patients in the doctor–patient relationship and ensures the effective practice of medicine. The article provides an insightful evaluation of the Belgian doctrine of professional secrecy, duty of assistance and communication between health care professionals under various relevant legislative frameworks. The article finds that although current Belgian legislation is unclear as to whether health care professionals or patients have a duty to disclose genetic information to at-risk relatives or such a duty of care, growing international trends may inform the interpretation and analysis of Belgian law in these settings and offer guidance.

On highlighting international precedent in this area, the article notes that in France, health care professionals are legally obliged to inform the patient beforehand about the risks of not disclosing a serious genetic risk to relatives. The relevant legislation in France imposes an express duty on a patient to inform their family members of their genetic risk in circumstances where there are preventative measures or treatment options available. In contrast, in Australia, legislation allows medical professionals to disclose genetic information to relatives in circumstances where they reasonably find such disclosure to be necessary to prevent or mitigate a serious threat to the life, health or safety of the relatives. The law requires that medical professionals inform their patients of the possibility during the informed consent process in advance of the genetic testing. In the UK, recent case law shows a judicial willingness to recognise an expanded duty on the part of medical professionals in these circumstances. The authors examine the landmark UK case of *ABC v St George’s NHS Trust* [2], which introduces a novel duty on medical professionals. This judgment establishes for the first time in UK law that health care professionals owe a legal duty, not only a professional obligation, to balance the rights and interests of at-risk individuals, such as a genetic relative, with those of a patient who has refused consent to disclosure of confidential information.

The international insights provided by this article are informative. Although acknowledging varying degrees of responsibility and duties on the part of medical professionals and patients, the position in these jurisdictions suggest a growing trend towards recognising a duty on the part of medical professionals to balance the interests of patients and relatives and in certain circumstances to disclose information of genetic risks to relatives, where such disclosure would avoid or mitigate harm to their life, health or safety. The authors adopt these insights to inform the conclusion that although Belgian law is unclear on the permissibility of disclosure of genetic information without patient consent, a duty to inform relatives of their genetic risk may be justified in certain cases.

## Conclusion: where next for law and policy in this area?

As documented in this article, the shared, familial nature of genetic information leads to situations where medical professionals are in a position to reveal test results that are relevant for the individual patient, as well as close family members [3]. In medical settings, non- consensual disclosure of genetic risk presents a range of particularly complicated challenges for the medical professional and the individual patient in question and highlights a tension between the duty of confidentiality and the potential avoidance of harm to others, through disclosure of risk. This presents practical concerns and competing interests, in ascertaining who the “patient” is [4]. It also creates challenges in determining the responsibilities of medical professionals (and patients) in these circumstances, as well as the extent to which family rights to genetic information might be deemed paramount to individual privacy rights, or where individual rights may be placed at the forefront.

If medical professionals have a responsibility to a patient’s family members, many questions remain as to the extent of that duty and what exactly the medical professional must do to fulfill such duty. In this regard, there are a number of considerations that medical professionals will need to take into account. The question of how serious the genetic condition is, as well as the varying degrees of genetic risk will all impact on the extent of any duty. If a disease is potentially fatal, and carries serious risk, the advantages in disclosing such risk may take precedence over the right to confidentiality and be reasonable in terms of preventing foreseeable harm [5]. Where a disease can be treated or prevented (for example, through monitoring or lifestyle alterations), disclosing a relevant genetic risk may be justified, to facilitate preparedness as well as prevention of disease [6]. Similarly, for conditions or diseases that have no cure, a sense of preparation (including psychological or financial) is also sometimes desirable in light of the seriousness of the disease.

The emergence of genetic medicine and the challenges presented require a the consideration of multiperspectives and nuanced approach to the medico-legal issues arising in this area. As acknowledged by the authors, further research on this topic is required by a multidisciplinary selection of stakeholders, including legal scholars, ethicists, social scientists and medical experts. Informed consultation with patient groups, families and health care professionals is also desirable to ensure a more informed and sophisticated understanding of the questions and dilemmas arising in this area. This article will spark much interest in the field and provoke further consideration, debate and discussion on the many issues in this area.
